# Progress in Medicine

**Published:** 1897-12-04

**Authors:** 


### Progress in Medicine.

### DISEASES OF THE DIGESTIVE ORGANS.

**The Liver** (*continued from page* 155).—Austin Flint,[28] in claiming priority in the discovery of stercorin, lays down that it is a definite chemical body and not impure cholesterin, from which, however, it is derived during the passage of the latter down the intestinal canal. Stercorin forms acicular crystals of an invariable type, as well as definite chemical compounds. The occurrence of cholesterin in the blood in excess of 0·75 parts in the thousand is of grave significance where certain liver diseases, such as cirrhosis, are in question, since the elimination of cholesterin, like that of urea, is one of the chief functions of the liver. Cholesteræmia may be a cause of those grave nervous symptoms without jaundice which are sometimes met with. Moreover, its ready combination with bromine may be the key to the action of bromides in epilepsy; indeed, there are few other substances with which it will combine, but its quantitative estimation in the blood is not a matter of difficulty to an ordinary chemist. A new cholagogue drug, a pure oleate of soda, called eunatrol, has been brought out of late, and, according to F. Blum,[29] is of high value, especially in biliary colic, when it reduces the attacks and causes the passage of many small stones and much gravel. Sixteen grains in pills are taken twice daily for a length of time, Rasmussen[30] recommends for the detection of bile pigment that a drachm of ether should be added to an equal quantity of urine, and shaken up with a few drops of iodine tincture; a brilliant green colour appears in the urine on standing if bile pigment is present. Among other supporters of the microbic origin of cirrhosis we must reckon Van Heukelom,[31] who refers it to bacterial poisons and others produced in the intestinal tract, while alcohol does not at any rate play a direct part in its production. He gives an exhaustive account of the experimental work done on the subject, and concludes that the cirrhotic state does not depend on cellular degeneration and necrosis. Thiemich,[32] too, has studied the livers of infants dying from gastro-enteritis. In some twenty out of thirty cases fatty degeneration had taken place. He thinks this was due to an intoxication from bacterial and other poisons acting on the cells of the organ. No alcohol had been given during the illness of the patients.

Another case of chylous ascites is reported by Apert,[33] which is remarkable for the absence of fat globules and a very small amount of fat at all. The serum in other parts of the body was clear, so that local causes would seem to have been at work, but no cancer or pressure on the duct or bacterial action could be made out. The pathology of tropical liver abscess following amœbic dysentery is fully discussed by Messrs. Howard and Hoover.[34] The ulcers in the intestines are deep with overhanging irregular edges, and frequently abscesses form in the submucosa, which may burst through into the peritoneum. In fatal cases of amœbic dysentery abscesses occur in the liver in from 20 to 60 per cent., and most commonly in the chronic forms of the disease. The abscess is usually on the upper surface of the right lobe. Besides a central area of soft necrotic tissue, there is a zone of gelatinous material and an outer one of fibrous tissue, and outside the abscess proper patches of necrosis showing liver cells may be found, due to toxins absorbed from the ulcerated intestines. In pure amœbic abscesses there is a striking absence of leucocytes, since the amœbæ have no positive chemotaxic action on them. Where mixed infection exists there is, however, leucocytosis. When rupture takes place into the lung, if adhesive inflammation has sealed up the pleura, a fresh abscess may form there, and may open into a bronchus, accompanied by chronic interstitial pneumonia. The expectoration may show large numbers of amœbæ. Dreschfeld[35] describes a case where an abscess formed in the left lobe, which is comparatively rare. Though it passed to a fatal termination in ten weeks, no rigors occurred. The physical signs on admission were those of fluid in the left pleura, but later on dulness was accompanied with increased vocal resonance and fremitus, tubular breathing, and pectoriloquy over the left lung, which after death was found to be the seat of a large secondary abscess.

**Appendicitis.**—Abbe[36] recommends that, when an appendix has been removed, it should be injected with 95 per cent. alcohol, ligatured, and immersed 24 hours in alcohol of the same strength. A longitudinal section will show an unsuspected number of ulcers, strictures, and other lesions. The development of the disease, he finds, begins with a catarrhal inflammation of the mucous membrane, narrowing of the calibre, and then strictures. The desquamating epithelium is thus retained, together with any particles of fæcal matter, and compressed into "concretions." Finally, complete obstruction, with distension and abscess, follows. Occasionally the disease may arise otherwise from internal ulceration or acute flexion. T. B. Beaver[37] recognises the occasional invasion of microbes through the lymph channels when the circulation of the organ is interfered with; but he thinks that the invasion more usually follows an erosion of the mucous membrane by some concretion which is moulded and rubbed by the contractions of the appendix. Inflammatory action follows both in the walls and outside of them, the latter producing "the limiting membrane," but in the vast majority of cases this membrane does not include the entire organ. Thus the base or the tips may be left free, and perforation may occur at those points. More or less obliteration of the lumen in quiescent cases is followed by spasmodic efforts of the organ to empty itself as its contents increase, leading frequently to rupture later on. As many of the abscesses contain bacillus coli, an attempt has been made to diagnose the presence of pus formation by Widal's method. Active cultures of the colon bacillus are found to be rendered immobile and slightly clumped by serum from cases where abscesses exist. Further research, however, is needed on this subject. Moizard[38] refers to the difficulty of diagnosis in hysterical subjects, who may simulate an appendicitis, but generally refer the pain to near the costal border, and present other deviations from the usual symptoms. Appendicitis in children generally follows gastro-intestinal disorders. He admits the occasional occurrence of typhlitis, which shows a slower onset and less severe symptoms. Dieulafoy,[39] admitting catarrhal and other forms of typhlitis, denies its origin from fæcal obstruction. When this does occur it is only secondary to appendicitis. He claims that the only treatment of the latter disease is by operation at the right moment, to which Dumontpallier replies that recovery in 90 per cent. of all cases is attained without it. When this disease was first studied most of the cases were found in males, but this appears to be partly because the symptoms in women are so often referred to uterine disorders. Fowler[40] points out that appendicitis has a more acute onset than diseases of the adnexa, the pain is more acute and radiating, pyrexia and vomiting are more frequent, muscular rigidity more common. The maximum tenderness is above the anterior spine, in adnexal disease it is more often below it, and on examination per vaginam the tender points in the latter diseases are easily made out, and pain is caused by moving the uterus. Besides, there may be a history of menstrual disturbances to aid the diagnosis.

The *Lancet*,[41] in reviewing the triumphs of surgery in appendicitis, refers to another side of the picture given by Kleinwächter from the practice of Professor Biermer before surgical interference was accepted. Out of 112 cases of perityphlitis only two died; all the rest recovered under treatment by complete rest, opium persistently used, ice bags, and very scanty fluid diet. Relapses after leaving the hospital occurred in 24 per cent. out of 70 cases, of which two were fatal, but none occurred later than two years after the first attack. Whilst a true comparison with cases under recent treatment is difficult, and the danger of rejecting surgical aid is great, these figures are not without importance. A disadvantage often felt at present is the hindrance to surgical diagnosis which the use of opium entails. Hence its almost complete disuse. Biermer's treatment not only showed a low mortality, but appeared to favour resolution and avert suppuration in many cases.

28 Med. Rec., June 6. 29 Therapist, April 15. 30 Hospit. Tidendes, p. 1,200, 1896. 31 Inter. Med. Mag., May. 32 Amer. Journ. Med. Sc., June. 33 Brit. Med. Jour., May. 34 Amer. Jour. Med. Sc., Aug. 35 Med. Chron., July. 36 Med. Rec., July 10. 37 Amer. Journ. Med. Sc., Aug. 38 Med. Week, April 9. 39 Med. Week, May 14. 40 Med. Chron., Aug. 41 Lancet, May 29.

### DISEASES OF THE LUNGS.

**Pneumonia.**—Tyson[1] states that, though the various types of pneumonia are pathologically almost identical, yet clinically they can be distinguished, and it is important so to do on account of their differing prognosis and treatment. The following kinds certainly exist: Pneumonia of influenza—In this form the consolidation is not, as a rule, confined to one lobe; it begins in small patches which coalesce. Often both lungs are involved. The signs of bronchial breathing and bronchophony are not well marked, but crepitant râles are prominent. The cough is troublesome, and the sputum, which is often profuse, does not possess the ordinary stickiness and rusty colour. Septic pneumonia—The cause is not always easy to discover, though occasionally it may follow what is evidently a septic disease—*e.g.*, erysipelas. The consolidation is patchy; and, whilst one patch is clearing up another may be appearing elsewhere. The physical signs are not well marked. The temperature follows a septic course, and the general appearance of the patient is one of blood-poisoning. There is no sputum; the disease is chronic in its course, and fatal in prognosis. Epidemic pneumonia—There is a rapid rise in temperature, and the constitutional disturbance is out of all proportion to the evidence of local pulmonary disease. Relapse is common, mortality is high. Convalescence usually begins by crisis on the seventh day. The disease appears to be a constitutional infection—a pneumonic fever. Typhoid or asthenic pneumonia comes on in diseased conditions. The pneumonia begins gradually and is never sharply defined, and is consequently frequently overlooked. The symptoms of rigor, pain, and rusty sputum are absent, and there appears to be no crisis. The pneumonia of the aged differs from the preceding types. It comes on insidiously, and is accompanied by a general body-weakness which may overshadow the lung mischief. The temperature is but little varied, often not above 100 deg. to 101 deg. F. The physical signs of dulness bronchial breathing, and crepitation are all present The prognosis is bad. In alcoholic pneumonia it is the delirium which is the prominent symptom, whilst the temperature is not high, and the expectoration is profuse and dirty in colour. The prognosis is bad.

Relapsing pneumonia is another type of pneumonia which may be clinically differentiated, and a case of this kind has recently been described by Oliver,[2] who says that a true relapse must be distinguished from the phenomena which occur in so-called migratory pneumonia, where successive portions of the lung are invaded, whilst the physical signs disappear from the parts first affected. In a true relapse there is a period of complete apyrexia, accompanied by advancing resolution, which is followed by a fresh rigor, rusty expectoration, crepitation, and a well-defined period of pyrexia.

The treatment of pneumonia may be of two kinds—radical, or an endeavour to destroy the germ and its toxin, or symptomatic, or an endeavour to combat the evil symptoms produced by these. We have recently had attempts at the production of an anti-toxin, of which some account will be given later; but the following hints in regard to the symptomatic treatment, by Foxwell,[3] are of service: The food should be light and unstimulating; milk only is usually enough, and in quantity from two to three pints—not more; thirst should not be allayed by milk, but by water given without stint; vomiting, constipation, and anorexia may follow the administration of milk at irregular intervals merely to allay thirst. The best routine treatment is to give at the onset a moderate calomel purge, of two to five grains, followed next morning by a saline draught. For the first three to five days the calomel should be continued every four hours in doses of one-twelfth to one-sixth of a grain. It is found that this does not depress, but is often invaluable by keeping the glandular system in free action. Occasionally the disease is ushered in by an intense and widespread pulmonary hyperæmia, which overthrows the right heart, so that the patient lies gasping and livid with distended veins in the neck. The only treatment for this, which is of any service, is venesection to the amount of, say, half a pint. The good effect is instantaneous, though not always lasting. Morphine is practically the only sedative for the intense pain which sometimes results from an accompanying pleurisy. Continuous high tension sometimes occurs, and is of serious import, because it so embarrasses the heart. To counteract this, some vasomotor dilator, such as nitrites or erythro-tetra-nitrate, may be given, and phenacetin for the headache which so often accompanies this high tension. Hyperpyrexia is best treated by the direct application of cold, and the form of this which is the most comforting is the cold pack. A sheet is wrung out in ice-cold or slightly warmer water, and wrapped round the patient. This may be reapplied as often as every half-hour. The feet and hands must be carefully watched, and stimulant administered if they become cold or blue. Hyperpyrexia may also be treated by a cold bath combined with rubbing during immersion, as proposed by Brand, and recently again advocated by Bishop.[4] Insomnia arising during the course of the disease, or persisting from the beginning, is a very serious matter, and if not due to any removable cause, such as flatulence, a loaded rectum, too little water, or too much food, is best treated by cold packing, and, if absolutely necessary, by morphine hypodermically. Alcohol, too, is often a good hypnotic. Cardiac exhaustion should be carefully watched for from the third day onward, and is best treated by strychnine given hypodermically, and by alcohol. Neither digitalis nor strophanthus is much to be depended on.

Bishop advocates the administration of bromides in large quantities from the very first in alcoholic patients, to lessen the danger of delirium. He admits that they do little good if delirium is once thoroughly established, but says they may advantageously be given as a preventative measure.

Washburn[5] has recently prepared an anti-pneumococcic serum in large quantities from the blood of a pony. The starting-point of the investigation was the observations of the Klemperers and others that the blood serum of rabbits immunised to the pneumococcus possessed the property of protecting other rabbits against infection with this micro-organism. The pony was repeatedly injected with graduated doses of pneumococci, and the blood serum, after some interval of time, was obtained. It was found that it possessed the power of protecting animals against infection when injected either at the same time or subsequently. Washburn then treated two patients suffering from pneumonia with this serum, in doses of 20 cb. cms. In one case the injection was followed on each occasion by a fall of temperature; but in neither case could it be said that the serum brought about a crisis in the disease. He advocates injection twice a day with the above-mentioned dose until the patient is convalescent.

Further cases submitted to this treatment have been published by Cooke[6] and by Harnett.[7] In the former case, which was probably one of pneumonia, complicating typhoid fever, the injections were generally followed by some degree of subjective improvement, though no marked effect on the physical condition of the lung or the temperature was observed. In the latter case the injections were followed on each occasion by a fall of temperature, and seemed to be influential in bringing about his recovery.

[1] Lancet, June 26, 1897. [2] Lancet, Sep. 4, 1897. [3] Treatment, Aug. 26, 1897. [4] Med. Record, Aug. 14, 1897. [5] British Med. Jour., Feb. 27, 1897. [6] Brit. Med. Jour., May 22, 1897. [7] Brit. Med. Jour., May 22, 1897.

